# Tuning the Phase Composition of Metal–Organic Framework Membranes for Helium Separation through Incorporation of Fullerenes

**DOI:** 10.1021/jacs.3c03362

**Published:** 2023-06-23

**Authors:** Jiuli Han, Haoyu Wu, Hongwei Fan, Li Ding, Guangtong Hai, Jürgen Caro, Haihui Wang

**Affiliations:** † Beijing Key Laboratory of Membrane Materials and Engineering, Department of Chemical Engineering, Tsinghua University, 100084 Beijing, China; ‡ College of Chemical Engineering, Beijing University of Chemical Technology, 100029 Beijing, China; § Institute of Physical Chemistry and Electrochemistry, Leibniz University Hannover, 30167 Hannover, Germany

## Abstract

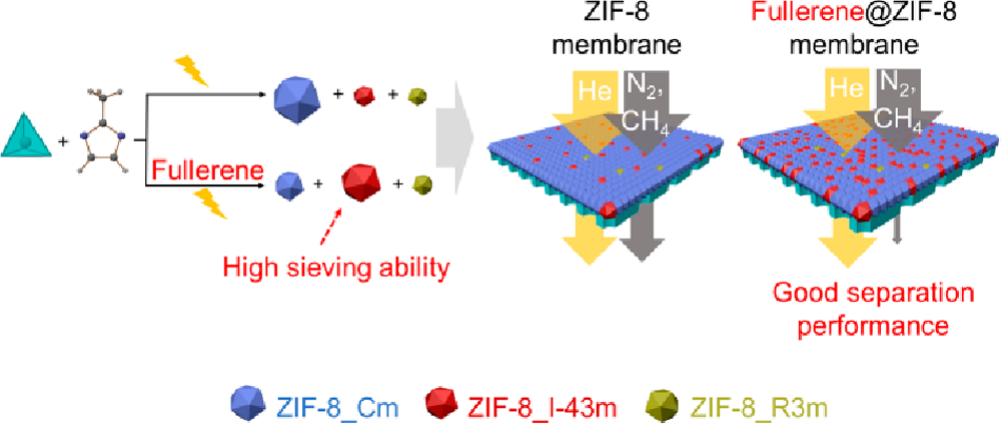

Metal–organic framework (MOF) membranes have attracted significant research interest in gas separation, but efficient helium (He) separation remains a challenge due to the weak polarizability of He and the intrinsic pore size flexibility of MOFs. Herein, incorporated fullerenes (C_60_ and C_70_) were used to tune the crystallographic phase composition of ZIF-8 membranes, thus creating small and fixed apertures for selective He permeation. The fullerene-modified ZIF-8 (C_60_@ZIF-8 and C_70_@ZIF-8) membranes contain about 20% of the rigid-lattice ZIF-8_I-43m phase and have been prepared as 200–350 nm thick supported layers through electrochemical synthesis. They show a significantly enhanced molecular sieving for He/N_2_,CH_4_ together with a satisfactory He permeance of >200 GPU. Specifically, the He/N_2_ selectivity of the C_70_@ZIF-8 membrane is up to 30.4, which is much higher than that of the fullerene-free ZIF-8 membrane (5.1) and nearly an order of magnitude higher than those of other reported He-selective MOF membranes. A continuous long-term gas permeation test over 780 h under dry and humid conditions proved the excellent stability of the fullerene-modified ZIF-8 membranes. The general validity and versatility of the proposed strategy for MOF membrane preparation are also demonstrated by the enhancement of the separation performance of a fullerene-modified ZIF-76 membrane.

## Introduction

The noble gas helium (He) is widely applied in diverse fields including magnetic resonance imaging, balloon ride, leak detection, welding, etc.^1,2^ He is one of the most important specialty gases for manufacturing semiconductors and electronic devices.^3,4^ As a rare gas on Earth, He usually exists in the atmosphere, natural gas, synthetic ammonia exhaust tail gas, and geothermal water in low concentrations.^5−7^ Currently, He production mainly depends on extraction from natural gas by cryogenic distillation and pressure swing adsorption, which is technologically challenging and always energy-intensive. A membrane-based separation technique has been proposed as a promising alternative to these conventional processes due to the low energy consumption, small CO_2_ footprint, and simple operation.^5^ The polyimide-based hollow fiber membrane SEPURAN Noble of Evonik has been recently commercialized for the He recovery from natural gas.^8^ Developing advanced membrane materials for efficient He separation and purification has been of continuous interest in the academic and industrial community since the existing polymeric membranes usually encounter a trade-off between permeability and selectivity and simultaneously suffer from plasticization and swelling.^2,9,10^ Molecular sieving membranes with ordered and tunable ultramicroporous structures are desirable for selective gas separation.^11,12^

As an emerging crystalline nanoporous material, metal–organic frameworks (MOFs) possess well-defined apertures and cavities, showing great potential in membrane-based gas separation.^13,14^ Nevertheless, the reported MOF membranes usually exhibit a low selectivity for He/N_2_ and He/CH_4_ (typically <3).^15,16^ The main reasons are the mismatched sieving size and the intrinsic aperture flexibility of MOF structure, causing a weak molecular sieving effect.^11,17^ For example, the flexible Zn–N bond and the swinging organic ligand in ZIF-8 will enlarge its effective aperture size to 4.0–4.2 Å, which is beyond the molecular-selective region for the selective separation of He from a mixture with N_2_ and CH_4_.^17−20^ Besides, although He has a tiny kinetic diameter of about 2.6 Å, its low polarizability leads to weak adsorption in MOFs, thereby further increasing the difficulties for its adsorptive separation.^21,22^ Approaches including metal ion and/or organic ligand replacement,^18,23−26^ electrochemical synthesis,^27^ and rapid heat treatment^28^ have been explored to tune the pore size and flexibility of MOF membranes. Among them, electrochemical synthesis is a simple and rapid method to fabricate ultrathin and defect-free MOF membranes.^29^ Simultaneously, this method can strengthen the rigidity of the MOF framework through restricted framework flexibility which benefits the separation of bulky gas pairs (e.g., C_3_H_6_/C_3_H_8_).^27^ However, for the separation of small gas pairs, such as He/N_2_ and He/CH_4_, it is important to enhance the molecular sieving by controlling the crystallographic phase composition of a MOF membrane.

The incorporation of nano entities into MOFs provides superior performance of the resulting host–guest composites for various applications.^30^ However, related studies on the MOF-based host–guest composites mainly focus on tuning the MOF cavity rather than the pore size. For example, an ultrathin UiO-67 membrane loaded with azobenzene (AZB) guest molecules was reported to separate H_2_/CO_2_; additionally the light-responsive AZB molecule performs trans–cis photoisomerization to control the separation performance.^31^ Ban et al.^32^ used an ionic liquid as a guest molecule to tailor the cavity of ZIF-8 thus increasing the CO_2_ separation performance of the polysulfone-based mixed matrix membrane. Li et al.^33^ reported a CuBTC/MIL-100 membrane with an enhanced H_2_ sieving ability through the incorporation of amorphous FeCl_3_ into the cavity of MIL-100. Owing to the nonregular molecular structure of these nano entities, it is difficult to precisely control the free volume inside a MOF cavity and to regulate the pore size between the adjacent cavities. Some guest molecules might also leach out from the MOF cavities, resulting in a changing separation performance. Fullerenes as a nonextractable guest in a MOF structure are hollow sp^2^ hybridized carbon cage macromolecules possessing unique characteristics, such as high electron affinity as well as good thermal and mechanical stabilities.^34,35^ Encapsulation of fullerene guests in MOFs has led to novel functional materials for catalysis,^36^ photoconductivity,^37^ and gas uptake.^38^ Notably, confined fullerene can give rise to structural contractions of host (i.e., MOF) through host–guest interactions.^39,40^

**Scheme 1:**
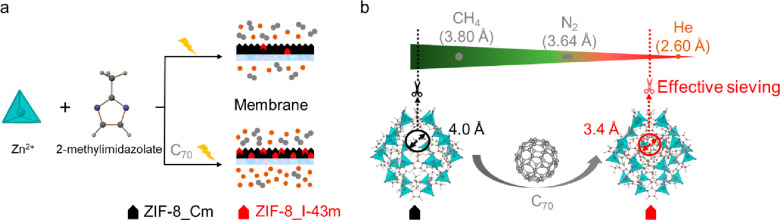
Illustration of the (a) Synthesis of Fullerene-Free and Fullerene-Modified ZIF-8 Membranes and (b) ZIF-8 Phase Transformation Due to Fullerene Incorporation Thus Generating the Rigid-Lattice ZIF-8_I-43m Phase with Small Pore Apertures Suitable for He Separation

In this study, we demonstrate the preparation of fullerene-tailored MOF thin-supported layers as sieving membranes by the *in situ* encapsulation of fullerenes during electrochemical MOF layer growth. Considering the large π-surface and rigid structure of fullerenes (C_60_ and C_70_), it is anticipated that the incorporated fullerenes C_60_ and C_70_ could induce a crystallographic phase transformation from the ZIF-8_Cm to the ZIF-8_I-43m phase, thus generating the rigid-lattice ZIF-8 membrane with small apertures and achieving an enhanced He selective separation, as shown in Scheme 1. It is well-known that the stiff phase has a high Young module of 29.2 GPa, reduces framework flexibility, and allows molecular sieving.^27^ As expected, the resulting fullerene@ZIF-8 membranes have significantly improved selectivities for He/N_2_ and He/CH_4_ separation compared to the ZIF-8 membrane and excellent long-term stability under dry and wet conditions. This strategy should open up a novel way to engineer effective pore sizes of MOF membranes for advanced molecular separations.

## Results and Discussion

The fullerene@ZIF-8 composite membranes were fabricated by electrochemical synthesis because of the short synthesis time of only dozens of minutes and the mild conditions.^41,42^ Mother solutions were first prepared by alternately adding the fullerene solution and the zinc acetate solution to the 2-methylimidazole solution (Figure S2a). After that, a conductive anodic aluminum oxide (AAO) disc as the substrate was vertically immersed in the mother solution, and the membrane was formed quickly on the AAO surface as a cathode under the electric field (Figure S2b).

**Figure 1:**
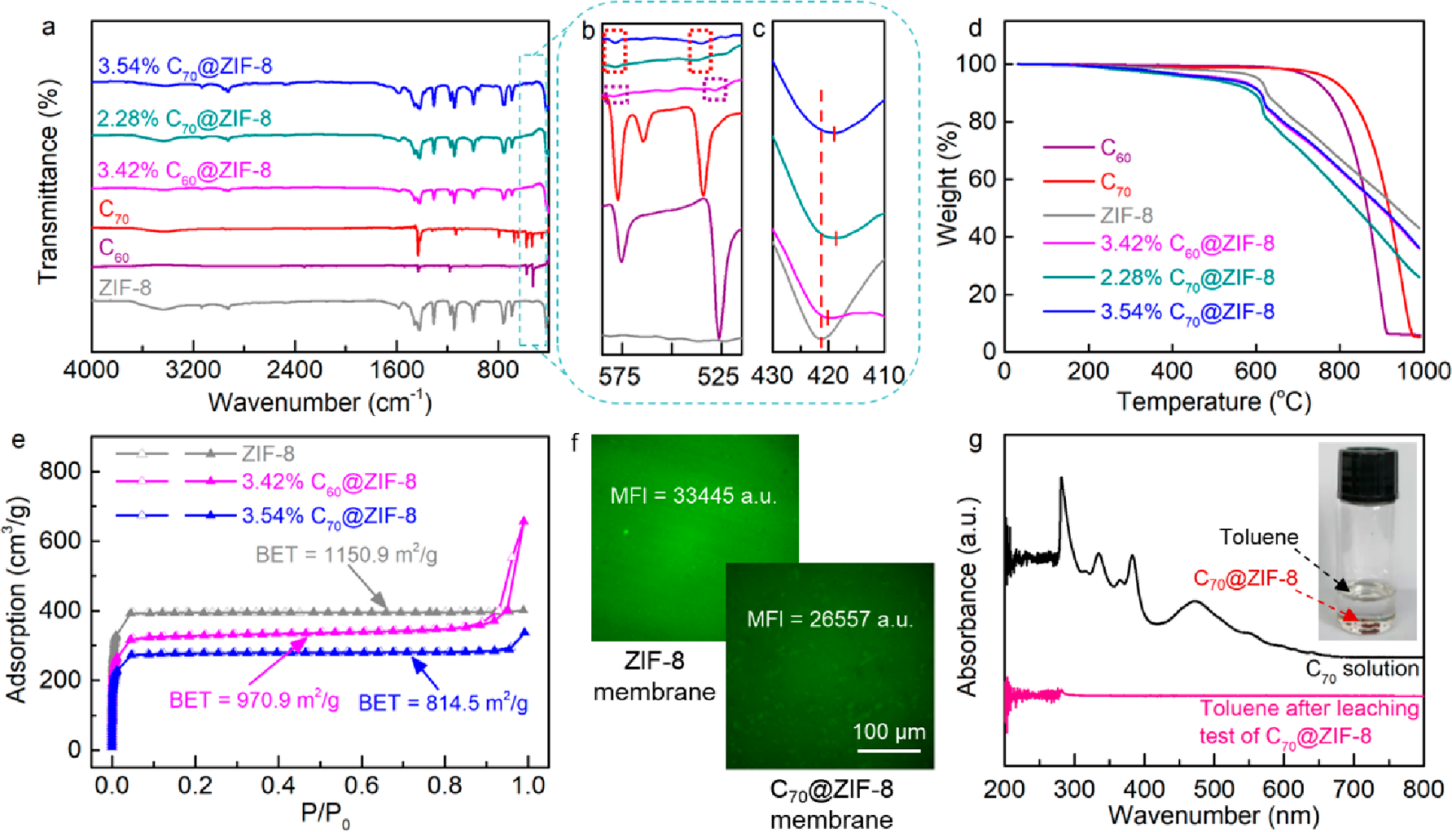
(a–c) FTIR spectra, (d) TGA curves, and (e) N_2_ adsorption (solid) and desorption (hollow) isotherms of the fullerene@ZIF-8 nanoparticles. (f) Fluorescence microscopy images of the surface (top view) of the pure ZIF-8 membrane and the C_70_@ZIF-8 membrane with the mean fluorescence intensity (MFI) value, and (g) UV–vis spectra of the C_70_ in toluene mixture and the toluene after leaching test of the C_70_@ZIF-8.

To verify the successful encapsulation of fullerenes into the ZIF-8 cavities, the C_60_@ZIF-8 and C_70_@ZIF-8 nanoparticles were collected from the sediment of the mother solutions and characterized by Fourier transform infrared (FTIR) spectroscopy, Brunauer–Emmett–Teller (BET), thermogravimetric analysis (TGA), and differential scanning calorimetry (DSC). From Figure 1a, the FTIR spectra of the C_60_@ZIF-8 and C_70_@ZIF-8 particles were found to be basically identical with those of ZIF-8. However, from the enlarged zone in Figure 1b, the fullerene signals at around 530 and 575 cm^–1^ become visible, which originate from the radial motion of carbon atoms in fullerene, and the intensities increased with the increase of the C_70_ content. Moreover, the ZIF peak at 421.4 cm^–1^ shifted to a longer wavelength for C_60_@ZIF-8 and C_70_@ZIF-8 (Figure 1c), indicating the increase in length and the weakening in the strength of the Zn–N bond. The slight decrease in the thermal stability of the C_60_@ZIF-8 and C_70_@ZIF-8 particles might be caused by the interaction of fullerene with the ZIF-8 framework, thus also supporting the successful incorporation of fullerenes (Figure 1d, Table S3). The DSC of ZIF-8 shows an endothermic feature at 624.9 °C which is shifted to lower temperatures of 621.7 °C, 617.7 °C, and 620.8 °C for 3.42% C_60_@ZIF-8, 2.28% C_70_@ZIF-8, and 3.54% C_70_@ZIF-8, respectively (Figure S5). From these experimental findings, it can be concluded that the ZIF-8 cavities were partially filled with fullerenes, as was already mentioned above. After the incorporation of C_60_ and C_70_, the N_2_ adsorption isotherm and BET surface area of ZIF-8 decrease (Figure 1e). The pore width distribution has two prominent peaks at around 10.0 and 12.7 Å (Figure S6), which shows the sizes of ZIF-8 cavities and micro defects, respectively. The position of the peak at 1.0 Å does not change after fullerene encapsulation, indicating that the encapsulation does not affect the cavity size of ZIF-8. Inverted fluorescence microscopy was adopted to study the fluorescence of the ZIF-8 membranes under study.^43,44^ The C_70_@ZIF-8 membrane has a lower mean fluorescence intensity (MFI) than the pure ZIF-8 membrane (Figure 1f), probably as a result of the C_70_ interaction with the nitrogen sites of ZIF-8. The leaching test of C_70_@ZIF-8 nanoparticles in toluene showed that the C_70_ molecules were not leached out since the color of the toluene solution did not change upon leaching, and also no characteristic peaks of C_70_ were detected by UV–vis spectroscopy (Figure 1g). This phenomenon is in accordance with the literature demonstrating a stable embedding of fullerenes inside the MOF cavities,^34^ which is beneficial to the stability of the membrane during long-term gas permeation.

**Figure 2:**
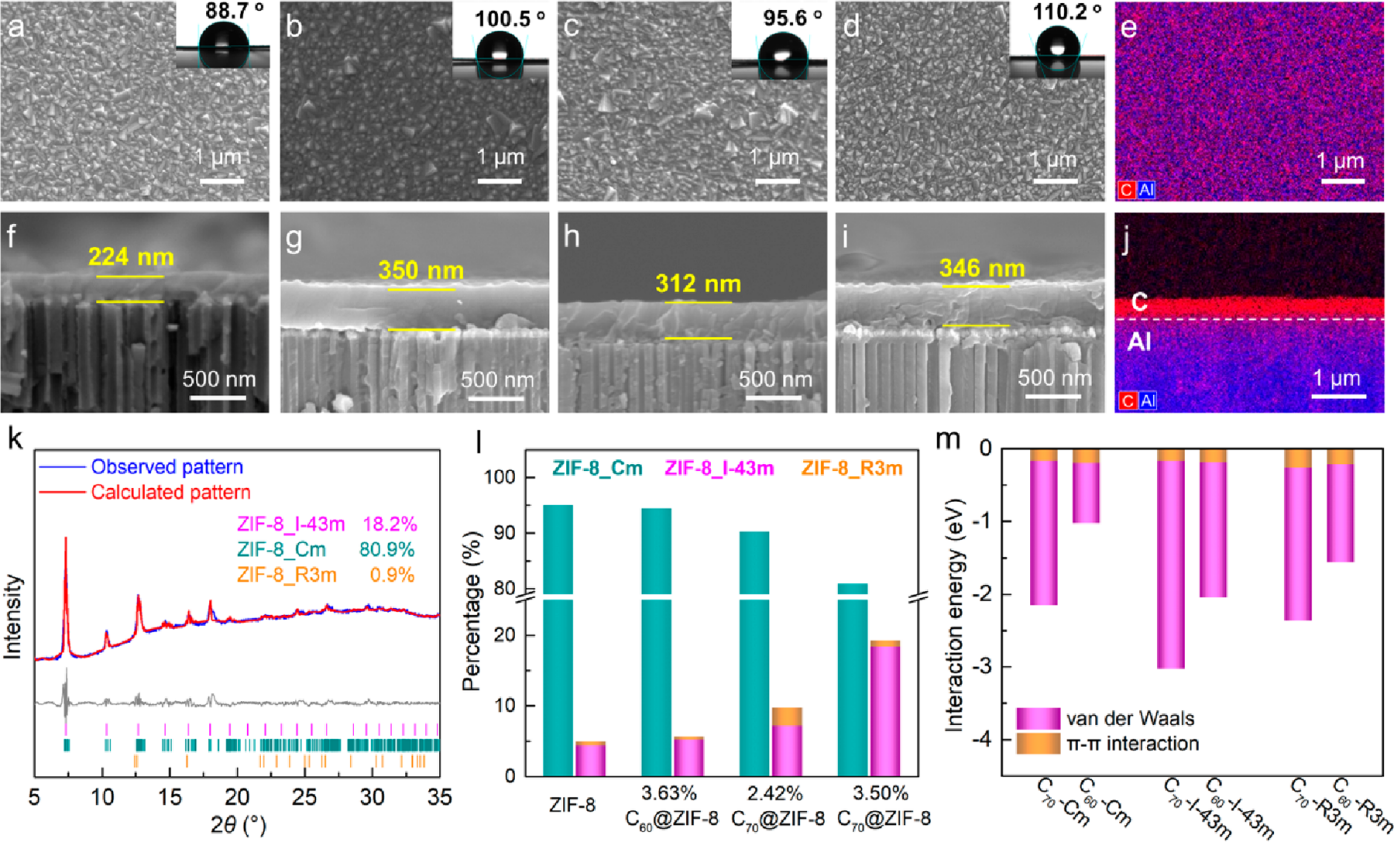
(a–d) Surface images and water contact angles and (f–i) cross-sectional images of the ZIF-8, 3.63% C_60_@ZIF-8, 2.42% C_70_@ZIF-8, and 3.50% C_70_@ZIF-8 membranes. (e) Surface EDS maps of C (red) and Al (blue) of the 3.50% C_70_@ZIF-8 membrane. (j) Cross-sectional EDS maps of C and Al of the 3.50% C_70_@ZIF-8 membrane. (k) Rietveld refinement of the XRD results for the 3.50% C_70_@ZIF-8 membrane. (l) Crystallographic phase percentages of the ZIF-8 membranes. (m) Calculated interaction energy between fullerenes and the lattices of the different ZIF-8 phases.

It follows from Figure 2a–d that the synthesized ZIF-8, C_60_@ZIF-8, and C_70_@ZIF-8 membranes have compact and continuous surfaces. Due to the strong hydrophobicity of the C_60_ and C_70_, and the increase of surface roughness (Figure S7), the fullerene@ZIF-8 membranes exhibit higher surface water contact angle values than the unloaded ZIF-8 membranes, which should be helpful for structural stability under humidity. The uniform element distribution of C on the surface of the 3.50% C_70_@ZIF-8 membrane implies its homogeneity (Figure 2e). The different membranes in this study are generally less than 350 nm (Figure 2f–i). Interestingly, the fullerene-modified membranes are thicker than the unloaded ZIF-8 membranes, suggesting that the fullerenes most probably accelerate the electrochemical growth rate of membranes by π–π and van der Waals interactions. From the cross-section (Figure 2j) it follows that the C element (red) is mainly concentrated on the top of the substrate, indicating that the selective ZIF-8 layer did not penetrate the AAO substrate. X-ray diffraction (XRD) patterns of the membranes exhibit the characteristic diffraction peaks of ZIF-8, suggesting no damage to the ZIF-8 structure through the introduction of C_60_ or C_70_ (Figure 2k and Figures S8–S10). The diffraction peaks of the C_60_ and C_70_ guests (Figure S11) are not observed in the membranes owing to the molecular-level dispersion and low content in the ZIF-8 matrix. Results of Rietveld refinement for XRD data show that about 20% of the 3.50% C_70_@ZIF-8 membrane structure is in the nonflexible ZIF-8_I-43m phase (Figure 2l), which is much higher than that of the fullerene-free ZIF-8 membrane (<5%). The main reason for this phenomenon is probably due to the structural changes of the unit cells around the cavity containing C_70_, thereby dramatically increasing the content of the rigid ZIF-8_I-43m phase. These changes in crystallographic phase composition were also observed in the 3.63% C_60_@ZIF-8, and 2.42% C_70_@ZIF-8 membranes. This finding suggests that the effective pore size of ZIF-8 has been reduced because the pore limiting diameters (3.4 Å, 3.1 Å) of the stiff ZIF-8_I-43m and ZIF-8_R3m phases are smaller than that (3.6 Å) of the usual ZIF-8_Cm phase,^27^ which will enhance the He separation performance of our membrane. Furthermore, we tried high-resolution transmission electron microscopy (HRTEM) to have a more intuitive observation of the fullerenes in the C_70_@ZIF-8 composites. However, these results were unsatisfactory, probably due to the low content of C_70_ in the ZIF-8 cavities and insufficient contrast between them (Figure S12).

To further elucidate the increase in the ZIF-8_I-43m phase after fullerene modification, the interaction between fullerenes and the different crystallographic phases of ZIF-8 was calculated by density functional theory (DFT) simulations (Figure 2m and Figure S13). Fullerenes interact with ZIF-8 through van der Waals and π–π interactions, and C_70_ shows a stronger interaction than C_60_. One of the reasons is the bigger molecular dimension and a shorter interaction distance. Compared with the other crystallographic ZIF phases, C_70_ has the strongest interaction with the ZIF-8_I-43m phase, which could rationally account for the increase in the percentage of this ZIF-8_I-43m phase after C_70_ modification. Higher content of this nonflexible ZIF-8_I-43m phase in the membrane enhances the molecular sieving ability of small molecules.

**Figure 3:**
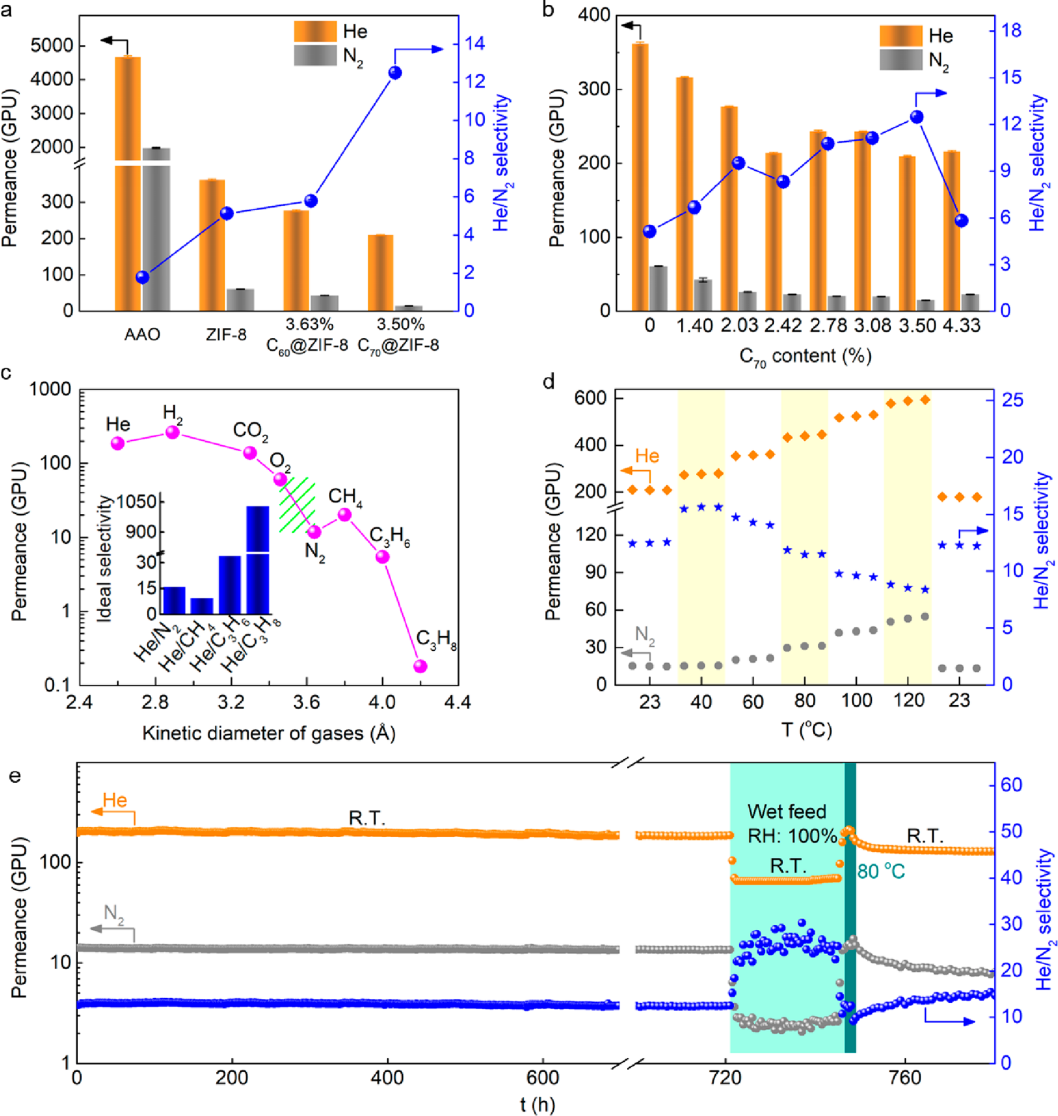
(a) He/N_2_ separation performances of AAO, ZIF-8, 3.63% C_60_@ZIF-8, and 3.50% C_70_@ZIF-8 membranes. (b) Effect of the C_70_ content on the He/N_2_ separation performance. (c) Pure gas permeance of the 3.50% C_70_@ZIF-8 membrane. The green area is the cutoff between different molecules. (d) Effect of temperature on the He/N_2_ separation performance of the 3.50% C_70_@ZIF-8 membrane. (e) Long-term stability of the 3.50% C_70_@ZIF-8 membrane.

Gas permeation measurements were conducted by placing the membrane inside a homemade module following the Wicke–Kallenbach method (Figure S14). He-rich natural gas mainly contains N_2_ and CH_4_ after removing impurities like acidic gases, heavy hydrocarbons, etc.^2^ Therefore, the single gases He, N_2_, and CH_4_ as well as equimolar binary mixtures of He with N_2_ and CH_4_ were used as feed for the separation tests at room temperature and 1 bar. It should be noted that the bare AAO support has only a negligible separation selectivity (1.8) for He/N_2_, and shows a very high gas permeance of above 4650 GPU (Figure 3a). As shown in Figure 3a, the fullerene@ZIF-8 membranes present an improved He/N_2_ selectivity compared with the fullerene-free ZIF-8 membrane. The 3.50% C_70_@ZIF-8 membrane has a higher selectivity than the 3.63% C_60_@ZIF-8 membrane due to the higher percentage of the ZIF-8_I-43m phase. The relatively low gas permeance can be explained by the increase of a certain gas transfer resistance due to the lattice stiffening. Further, we investigated the effect of the C_70_ content on the separation performance (Figure 3b). With increasing C_70_ content, first the gas permeance decreases and then levels off. This decrease of both the He and N_2_ permeability is due to an increasing concentration of the less flexible ZIF-8_I-43m phase but not an effect of an increasing cavity blocking, in view of the relatively low fullerene loading of only up to 3.50%.^45^ Meanwhile, the He/N_2_ selectivity gradually increases from 5.1 to 12.5, but then decreases to 4.3% C_70_. With the increase of the C_70_ load, there could be nonencapsulated C_70_ molecules as aggregated clusters at the boundaries of the polycrystalline ZIF-8 membrane, causing some defective pathways for gas transport in the resulting membrane. By compromise of selectivity and permeance, the 3.50% C_70_@ZIF-8 membrane was selected for further investigation.

The different single-gas permeances of the 3.50% C_70_@ZIF-8 membrane are shown in Figure 3c. The He permeance is 185.4 GPU, which is much higher than that of all other probe gases except H_2_ with 262.6 GPU. This phenomenon is likely attributed to the weaker adsorption and polarizability of He in ZIF-8 in comparison with H_2_, despite the similar diffusivities.^21,22^ A cutoff at around 3.5 Å can be observed, resulting from the smaller pores of the ZIF-8_I-43m phase due to lattice stiffening in the C_70_@ZIF-8 membrane. This phenomenon can be used to effectively separate He from more bulky molecules, and thus, the ideal selectivities of He/N_2_, He/CH_4_, He/C_3_H_6_, and He/C_3_H_8_ increase up to 15.8, 9.2, 34.1, and 1030, respectively. The ideal selectivity of He/N_2_ (15.8) is slightly higher than that of the mixed gas (12.5). The effect of temperature on the separation performance was also studied using equimolar He/N_2_ mixtures (Figure 3d). The He permeance increases gradually with increasing temperature, while the N_2_ permeance is almost unchanged in the low-temperature range (23–40 °C) but then increases due to the highly activated diffusion of N_2_ in comparison with He, which is confirmed by a higher apparent activation energy of N_2_ (17.01 kJ/mol) in comparison with that of He (9.10 kJ/mol) (Figure S15). As a result, the He/N_2_ selectivity first increases slightly and then starts to decrease at 60 °C. Damage to the membrane structure from the thermal decomposition of ZIF-8 and C_70_ can be rationally excluded, and the He/N_2_ selectivity of the C_70_@ZIF-8 membrane remains above 8 at 120 °C, indicating good thermal stability. Besides, the He/N_2_ separation performance can be recovered when the temperature drops again to room temperature. Similar phenomena were also observed in the separation of He/CH_4_ mixtures as the testing temperature varied (Figure S16). The He/N_2_ selectivities are higher than the He/CH_4_ selectivities, probably due to a high affinity of the membrane to hydrocarbons after encapsulation of fullerenes.^46^

In addition, long-term continuous operation was performed to explore the stability of the C_70_@ZIF-8 membrane. From Figure 3e, both the He permeance and the He/N_2_ selectivity show no obvious changes during the 720 h test. Under 100% relative humidity (RH), although both the He and N_2_ permeances decrease due to the competitive diffusion of water molecules, the He/N_2_ selectivity was increased to 30.4, which is more than two times that under dry conditions. This increase of He/N_2_ selectivity could be ascribed to the narrowed gas-transport channels by water molecules,^47^ thereby strengthening the sieving ability. It is worth noting that the former separation performance can be recovered after removal of the water. These results indicate the outstanding stability of the fullerene@ZIF-8 membrane under dry and humid conditions.

**Figure 4:**
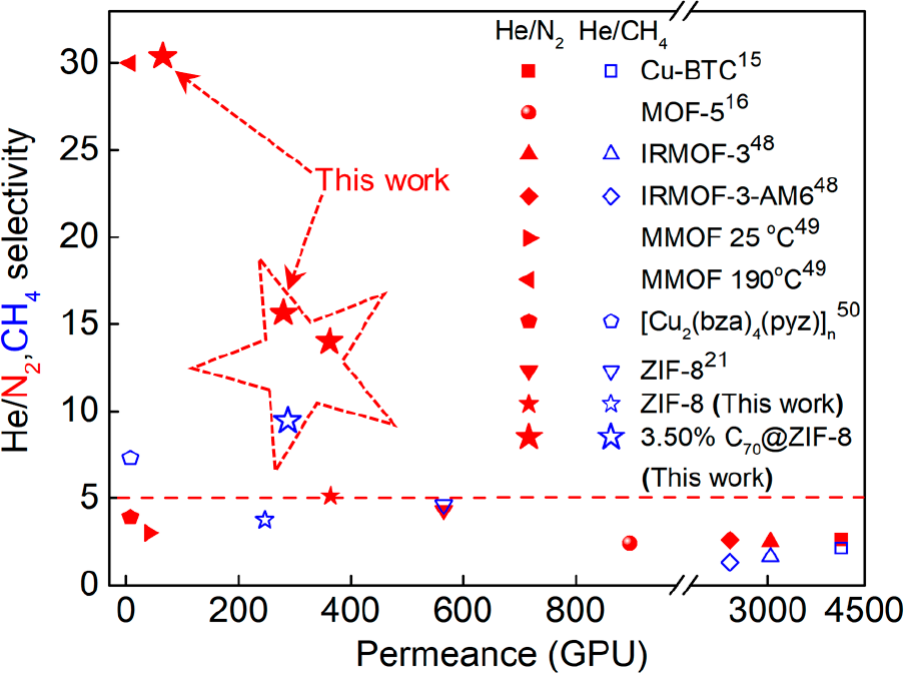
Selectivity of He/N_2_,CH_4_ as a function of He permeance of the fullerene@ZIF-8 membrane compared with literature data (for detailed information on the data points, see Table S4.).

As a benchmark of membrane performance, the selectivity of He/N_2_ and He/CH_4_ separations versus the He permeance for our fullerene@ZIF-8 membrane and other MOF membranes is illustrated in Figure 4.^15,16,21,48−50^ Most He/N_2_ and He/CH_4_ selectivities are below 5 (Figure 4, Table S4). By contrast, the He/N_2_ and He/CH_4_ selectivities of the 3.50% C_70_@ZIF-8 membrane are higher and can reach up to 15.8 and 9.2, respectively. Moreover, the He/N_2_ selectivity could be further enhanced to 30.4 under wet conditions.

To demonstrate the versatility of our fullerene-tuning strategy, we have successfully fabricated another type of fullerene@MOF membrane by encapsulating C_70_ in the ZIF-76 cavity with a diameter of 12.2 Å.^51^ The obtained C_70_@ZIF-76 membranes had uniform thicknesses of 396 nm (Figure S17). Moreover, the membrane surface also became more hydrophobic and rougher than before (Figure S18 and Figure S19). The C_70_@ZIF-76 nanoparticles collected from the sediment of the membrane synthesis were characterized by FTIR (Figure S20). The prepared membrane exhibits remarkably improved selectivities for He/N_2_ and He/CH_4_ compared with the fullerene-free ZIF-76 membrane (Table S5). This result further indicates the effectiveness and universality of phase composition regulation of MOF membranes by fullerene incorporation for enhanced He separation.

**Figure 5:**
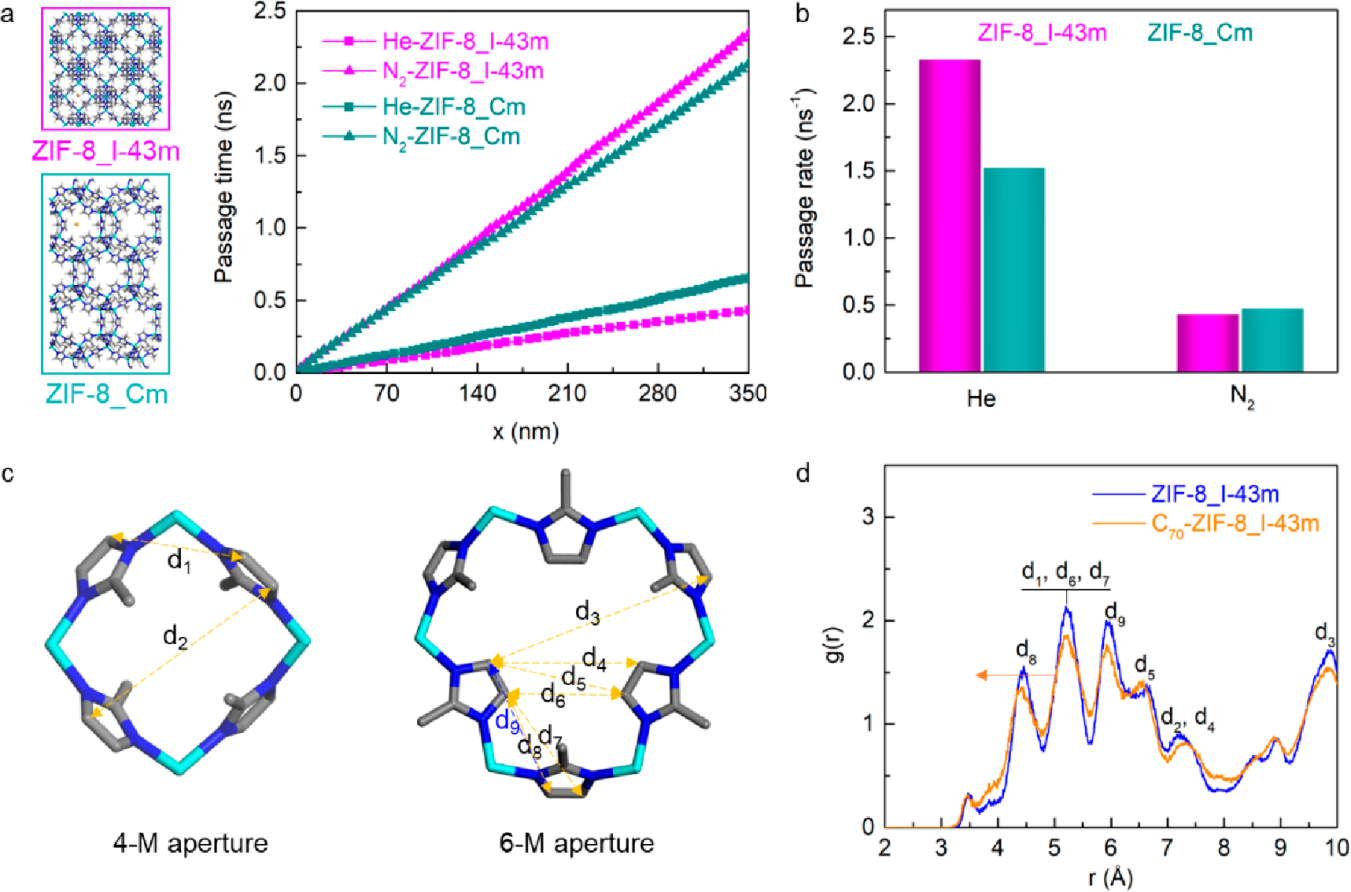
(a) Average passage time of He atoms or N_2_ molecules through a ZIF-8_I-43m or ZIF-8_Cm membrane with a thickness of 350 nm. (b) The passage rate of He and N_2_ through the membrane. (c) 4-M and 6-M apertures of the ZIF-8_I43m phase. (d) Distances between different carbon atoms at 25 °C.

For a deeper molecular understanding of the separation performance and the mechanism, simulations of He atoms and N_2_ molecules passing through the membranes were performed by using molecular dynamic (MD) simulations. The passing time is computed and is shown in Figure 5a. He atoms need a shorter time to penetrate through a ZIF-8_I-43m membrane than through a ZIF-8_Cm membrane, while the passage time of the larger N_2_ molecules through a ZIF-8_I-43m membrane is much longer since nitrogen needs lattice flexibility for passing. Therefore, ZIF-8_I-43m has a lower passage rate for N_2_ but a higher passage rate for He (Figure 5b), leading to high He/N_2_ selectivity. Additional simulations show that C_70_ affects the 4-M and 6-M apertures of the ZIF-8-I_43m phase (Figure 5c). The radial distribution function (RDF) of carbon atoms of ZIF-8_I-43m phase before and after C_70_ modification is given in Figure 5d, showing the most probable distances between the different carbon atoms; the values are listed in Table S6. For the 4-M aperture, its pore size is 2.8 Å,^52^ which is larger than that (2.6 Å) of the He atom allowing it to pass. In this aperture, *d*_2_ increases and *d*_1_ slightly decreases after C_70_ modification, enlarging the pores to increase He permeance but not enough for N_2_ molecules to pass through, thus leading to an increase in He/N_2_ selectivity. In the 6-M aperture, all distances become shorter except *d*_3_ and *d*_4_ after introducing C_70_, indicating that the aperture size decreases. This will similarly enhance the sieving ability of the membrane for He.

## Conclusion

In summary, we report the electrochemical synthesis of supported He-selective fullerene-modified MOF membranes. Due to the incorporation of fullerenes, a critical concentration of the more rigid ZIF-8_I-43m phase with a small pore aperture is formed, and the molecular sieving ability of the fullerene-modified ZIF-8 membrane for the selective separation of small molecules/atoms is enhanced due to lattice stiffening. The fullerene@MOF composite membranes exhibit He/N_2_ and He/CH_4_ selectivities higher than those of the unloaded ZIF-8 membrane, and they are also ultrastable under dry and wet conditions, as manifested during a continuous 780 h test. Given the excellent performance and successful fabrication of different fullerene@MOF membranes, this work will inspire the design and structural regulation of MOF-based gas separation membranes and other host–guest functional materials for various applications.

## Supporting Information Available

Experimental details on materials, characterization, gas separation test, supplementary characterization results, and performance. (PDF)
