# Supplementary material for: Tuning the Phase Composition of Metal–Organic Framework Membranes for Helium Separation through Incorporation of Fullerenes
Source: J Am Chem Soc. 2023 Jun 23;145(27):14793–801. doi: 10.1021/jacs.3c03362 (PMC10347541; doi:10.1021/jacs.3c03362)
Supplement: Supplementary file 1 — ja3c03362_si_001.pdf [file ja3c03362_si_001.pdf]

Supporting Information

Tuning the Phase Composition of Metal-Organic Framework Membranes for Helium Separation through Incorporation of Fullerenes

Jiuli Han,[a] Haoyu Wu,[a] Hongwei Fan,*[,b] Li Ding,[a] Guangtong Hai,[a] Jürgen Caro,*[,c] and Haihui Wang*[,a]

[a] Beijing Key Laboratory of Membrane Materials and Engineering, Department of Chemical Engineering, Tsinghua University, 100084 Beijing, China.

[b] College of Chemical Engineering, Beijing University of Chemical Technology, 100029 Beijing, China.

c Institute of Physical Chemistry and Electrochemistry, Leibniz University Hannover, 30167 Hannover, Germany.

Materials

Zinc acetate dihydrate (>99.99%, Aladdin), 2-methylimidazole (mIm, 98%, Aladdin), imidazole (Im, 99%, Meryer), 5-chlorobenzimidazole (cbIm, 97%, Meryer), 5-methylbenzimidazole (mbIm, 97%, Mreda), fullerene-C_{60} (99%, Meryer), fullerene-C_{70} (98%, Meryer), methanol (≥99.5%, Aladdin), toluene (≥99.5%, Beijing Tong Guang Fine Chemicals Company), porous anodic aluminum oxide (AAO, diameter of 18 mm, pore diameter of 40-70 nm, Pu-Yuan Nanotechnology Limited Company). All the chemicals were used as received without further purification.

Preparation of mother solutions and membranes

The fullerene@ZIF-8 membranes were prepared by the electrochemical synthesis method, which possesses the advantages of short reaction time and mild conditions.[1-2] Before the fabrication of the membrane, the mother solution needs to be prepared firstly. ZIF-8 mother solution was prepared by dissolving a mixture of 0.55 g zinc acetate dihydrate and 0.41 g 2-methylimidazole (mIm) in 50 mL methanol under ultrasonication for 10 min. The molar ratio of mIm/Zn^{2+} in the mother solution was 2:1.

For the preparation of fullerene@ZIF-8 mother solutions, due to the low solubility of the fullerenes in methanol, the fullerenes were firstly dissolved in toluene and then dropped into the ZIF-8 mother solution. A certain amount of C_{60} was placed into a 20 mL vial, and then a calculated volume of toluene was added to form a uniform solution with a C_{60} concentration of 1 mg/mL. Similarly, the solution of C_{70} in toluene was obtained. The solutions of C_{60} and C_{70} were purple and dark brown (Figure S1), and were marked as S-C_{60} and S-C_{70}, respectively. Then, 0.41 g mIm and 0.55 g zinc acetate dihydrate were separately dissolved in 25 mL methanol under ultrasonication for 10 min and the solutions were denoted as S-L and S-Zn, respectively. To make sure an efficient encapsulation of

fullerene in ZIF-8 cavity, S-Zn was added after the addition of the fullerene solution. As shown in Figure S2a, taking the C_{70}@ZIF-8 mother solution as an example, 300 μL S-C_{70} was first added dropwise into 25 mL S-L within 10 min under magnetic stirring. After that, 12.5 mL S-Zn was added dropwise into the mixture within 30 min. Next, 300 μL S-C_{70} was added dropwise within 10 min, and then followed an addition of another 12.5 mL S-Zn solution within 30 min. Finally, the system was continuously stirred for 1 h to obtain the mother solution. The detailed dropping parameters for preparing other mother solutions were given in Table S1. The prepared mother solutions were labeled as X μL $C_{60(70)}$@ZIF-8, where the X represents the value of the added total volume of the S-C_{60} or S-C_{70}. To determine the specific amounts of fullerenes in the fullerene@ZIF-8, the fullerene@ZIF-8 nanoparticles were separated from the mother solutions and added into 1 mL hydrochloric acid (1 M). When the framework of ZIF-8 was destroyed, 4 mL toluene was added to extract the released fullerenes (Figure S3). Then the UV-vis spectra of the extracts were measured using a Cary Series UV-Vis-NIR Spectrophotometer (Agilent) to obtain the intensity of absorption peak and the concentrations of the fullerenes were obtained using the calibrated UV-vis standard curves (Figure S4). The contents of fullerenes in the fullerenes@ZIF-8 nanoparticles were calculated using the following equations and the results were listed in Table S2.

$$C_1 = \frac{I - 0.0002}{0.1054} \quad \text{(Standard curve of } C_{60}\text{)} \tag{1}$$

$$C_1 = \frac{I + 0.0026}{0.0623} \quad \text{(Standard curve of } C_{70}\text{)} \tag{2}$$

$$M_2 = C_1(V_2 + V_3)\frac{V_1}{V_2} \tag{3}$$

$$C = \frac{M_2}{1000M_1} \times 100\% \tag{4}$$

$$O = \frac{(M_2 / M_{r(C_{60} or C_{70})})}{[(M_1 - M_2) / M_{r(ZIF-8)}]} \times 100\% \tag{5}$$

Note:

M_1: mass of the sample

V_1: volume of toluene used to extract fullerene

V_2: volume of part extract from V1

V_3: volume of pure toluene used to dilute the part extract (V_2)

I: intensity of the absorption peak

C_1: concentration of fullerene in V_2+V_3 obtained from the standard curve

M_2: mass of fullerene in the sample

C: the mass percentage of fullerene in fullerene@ZIF-8 nanoparticles

O: occupancy of fullerene in ZIF-8 cavities, namely the number of fullerene molecules per 100 ZIF-8 cavities

$M_{r(C60 \text{ or } c70)}$: relative molecular mass of fullerene

$M_{r(ZIF\text{-}8)}$: relative molecular mass of ZIF-8 cavity (The simplest molecular formula of ZIF-8 is $C_8H_{10}N_4Zn$ and its M_r is 227.58 g/mol. One cavity is composed of 24 zinc ions and the ions are shared by 4 cavities. Thus, one cavity actually possesses 6 zinc ions and the molecular weight of one ZIF-8 cavity is 227.58 g/mol×6=1365.48 g/mol)

For the content of fullerene in the membrane, ICP-MS was used to determine the molar amount of Zn^{2+} ($N_{(Zn^{2+})}$) because the ZIF-8 crystal growths on the AAO substrate and the value of M_1 is difficult to obtain. UV-vis was adopted to measure the molar amount of fullerene ($N_{(C60 \text{ or } C70)}$). The occupancy was calculated using the following equation and given in Table S2:

$$O = \frac{N_{(C_{60} or C_{70})}}{[N_{(Zn^{2+})}/6]} \times 100\% \quad (6)$$

Electrochemical synthesis of the fullerene@ZIF-8 membranes is illustrated in Figure S2b. A porous anodic AAO substrate was coated with Pt using a sputter coater (Quorum Q150R ES) at 20 mA for 300 s and then it was vertically immersed into the mother solution. The distance between the two electrodes is 5 cm. At room temperature, an electric field with a current density of 0.7 mA·cm^{-2} was exerted. After 20 min, the MOF layer was in situ grown on the surface of AAO

substrate. Finally, the formed membrane was kept at room temperature for 24 h and then dried at 80 °C in a vacuum oven for 12 h. The membrane was labeled as X% $C_{60(70)}$@ZIF-8, where X represents the fullerene occupancy in ZIF-8 cavities (Table S2).

Characterization. Fourier transform infrared (FTIR) spectra of ZIF-8, C_{60}@ZIF-8, and C_{70}@ZIF-8 nanoparticles were measured by Nicolet iS50 FTIR spectrometer with the range of 4000-400 cm^{-1}. Thermogravimetric analysis (TGA) and differential scanning calorimetry (DSC) curves of ZIF-8, C_{60}, C_{70}, C_{60}@ZIF-8, and C_{70}@ZIF-8 particles were performed using Mettler Toledo TGA/DSC 3^{+} Star System in N_2 with a heating rate of 10 °C/min. Micromeritics 3FLEX instrument was used to measure the N_2 adsorption and desorption isotherms at -196 °C. Before test, the samples were vacuumed at 80 °C for degassing. Density functional theory model was adopted to calculate the pore size distribution. Fluorescence properties of ZIF-8 and 3.50% C_{70}@ZIF-8 membranes were analyzed by an inverted fluorescence microscope Ti2-U (Nikon) platform equipped with an ELWD objective lens (40x, 0.6 NA). X-ray diffraction (XRD) patterns of the membranes were recorded under ambient conditions with a Rigaku MiniFlex600-C diffractometer with Cu Kα radiation. Calculations on the XRD patterns were performed with GSAS II software. Water contact angles on membranes were tested by sessile drop method with a Dataphysics OCA15EC optical contact angle device. Scanning electron microscope (SEM) images of the membranes were obtained by a JEOL JSM-7900F apparatus and all samples were coated with Pt before characterization. Surface roughness of the membrane was characterized by an atomic force microscope (AFM) using a Bruker Multimode 8 instrument. High-resolution transmission electron microscope (HRTEM) images of the ZIF-8 nanoparticles, ZIF-8 membrane, 3.54% C_{70}@ZIF-8 nanoparticles, and 3.50% C_{70}@ZIF-8 membrane were taken by a JEM-2010 instrument. The samples were dispersed in methanol under ultrasonication for 3 min. The prepared solutions were dropped on a carbon-coated copper grid

and then dried in a vacuum oven at 70 °C for 12 hours.

Gas permeation test. For a single gas permeation test, the prepared membrane was fixed in a module sealed with O-rings. A volumetric flow rate of 25 mL min^{-1} gas was applied to the feed side of the membrane, and the permeate gas was removed from the permeate side by Ar gas. Pressures at both the feed side and permeate side were maintained at 1 bar. A calibrated gas chromatograph (Agilent 8890) was used to measure the concentration of each gas on the permeate side. The gas permeance (P_i) is defined using the following formula (7).

$$P_i = \frac{V_i}{(P_{feed} - P_{perm}) \times A} \tag{7}$$

where P_i is permeance in GPU (1 GPU = 1 × 10^{-6} cm^3 (STP)/(cm^2·s·cmHg)), i represents the penetrating gas, V_i is the flow rate of gas i, P_{feed} and P_{perm} are the partial pressures of the gas i at the feed and permeate sides, separately. A is the effective area of the membrane. Ideal selectivity (S) is the ratio of the permeances of two permeating gases (i and j).

$$S = \frac{P_i}{P_j} \tag{8}$$

In the test of the mixed gas separation, the flow rates of the two gases (gas i and j) were 25 mL min^{-1}. The gas permeance was similarly calculated using the above equation (7). The actual selectivity of gas i and gas j is defined as:

$$\alpha = \frac{x_{i,\,perm} / x_{j,\,perm}}{x_{i,\,feed} / x_{j,\,feed}} \tag{9}$$

where $x_{i,feed}$ and $x_{i,perm}$ are the mole fractions of gas i in the feed and permeate sides.

Simulation methodology.

All the density-functional theory (DFT) computations were performed using the Cambridge Sequential Total Energy Package (CASTEP) based on the pseudopotential plane wave (PPW) method. Electron-ion interactions were

described using the ultrasoft potentials (USP). A plane-wave basis set was employed to expand the wave functions with a cutoff kinetic energy of 450 eV. For the electron-electron exchange and correlation interactions, the functional parametrized by Perdew-Burke-Ernzerhof (PBE), a form of the general gradient approximation (GGA), was used throughout. The van der Waals interaction was described using the DFT-D2 method that proposed by Grimme.

During the geometry optimizations, all the atom positions were allowed to relax. In this work, the Brillouin-zone integrations were conducted using Monkhorst-Pack (MP) grids of special points with the separation of 0.06 $Å^{-1}$. The convergence criterion for the electronic self-consistent field (SCF) loop was set to $5×10^{-7}$ eV/atom. The atomic structures were optimized until the residual forces were below 0.01 $eVÅ^{-1}$, and the cell parameters were also optimized with finite basis correction until the stress was less than 0.02 GPa.

Classical molecular dynamics (MD) was utilized to understand the effect of C_{70} on the structure of ZIF-8_I-43m phase before and after C_{70} modification. The system first undergoes a 500-step steepest-decent energy minimization. It was then run under an NVT ensemble (constant particle number, volume, and temperature), randomly selecting initial velocities. The temperature was maintained at 298 K by the Nosé-Hoover method for 500 ps with a time step of 1 fs. The Universal Force Field (UFF) was used for the bonded interaction between atoms, and the atoms are assumed charge-neutral.[3] Lenard-Jones potential was applied to the non-bonded interaction between a pair of atoms. The cutoff distance was 12.5 Å with a buffer width of 0.5 Å. During the simulation, the positions of the Zn atoms of the ZIF-8 membrane were fixed while other atoms remained movable as the ZIF-8 structure was considered partially flexible.[4-5] Periodical boundary conditions were applied to *x*- and *y*- and *z*-directions.

The radius distribution function (RDF) g(r) was computed to analyze the gate opening of 4M and 6M apertures of ZIF-8_I-43m phase with different structures.[5]

$$g_{ij}(r) = \frac{N_{ij}\left(r, r+\Delta r\right)V}{4\pi r^2 \Delta r N_i N_j} \tag{10}$$

Where r is the distance between atom *i* and *j*, N_{ij} is the number of atoms *j* around center atom *i* within a bin of Δr at a distance of *r*, *V* is the structure occupied volume, and N_i and N_j are the atom numbers.

Diffusion simulations were performed to investigate the diffusion behavior of He and N_2 molecules through ZIF-8 membranes. Simulation boxes with periodical boundary conditions were built with fixed structures of ZIF-8_I-43m phase, ZIF-8_Cm phase, and 20 randomly distributed He/N_2 molecules. NVT simulations were performed with a time-step of 0.2 fs. A constant force of 10 (kcal/mol)/Angstrom was applied to each molecule to facilitate the movement in the permeation direction. Times of He and N_2 molecules reaching 350 nm distance from their starting position were recorded (Figure 5a) and converted into passing rates (Figure 5b). All simulations were performed in LAMMPS (23 June 2022 version).[6]

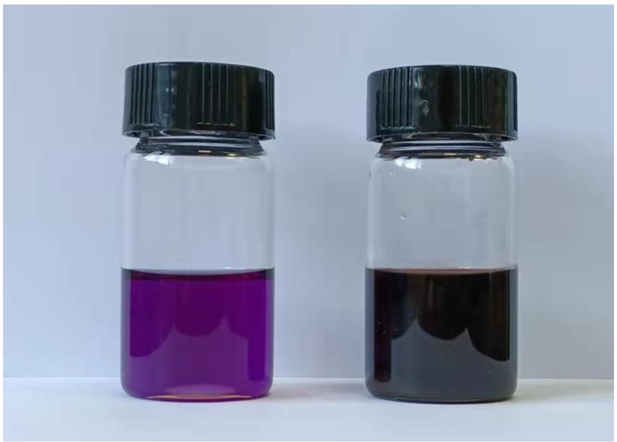

Figure S1. 1 mg/mL solutions of C_{60} (left) and C_{70} (right) in toluene.

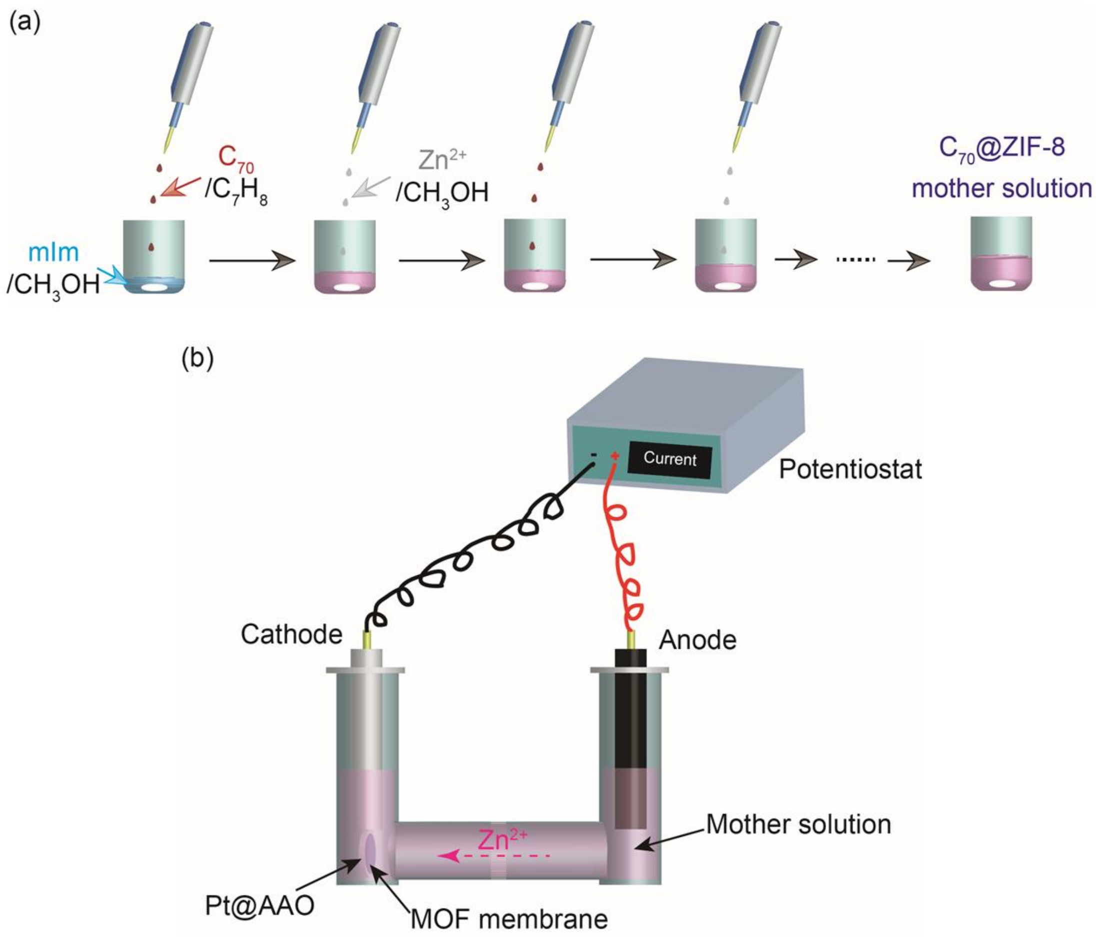

Figure S2. Schemes of the preparation of the fullerene@ZIF-8 mother solution (a) and membrane (b).

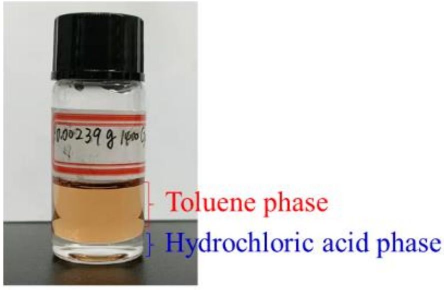

Figure S3. Extraction of the released fullerene with toluene.

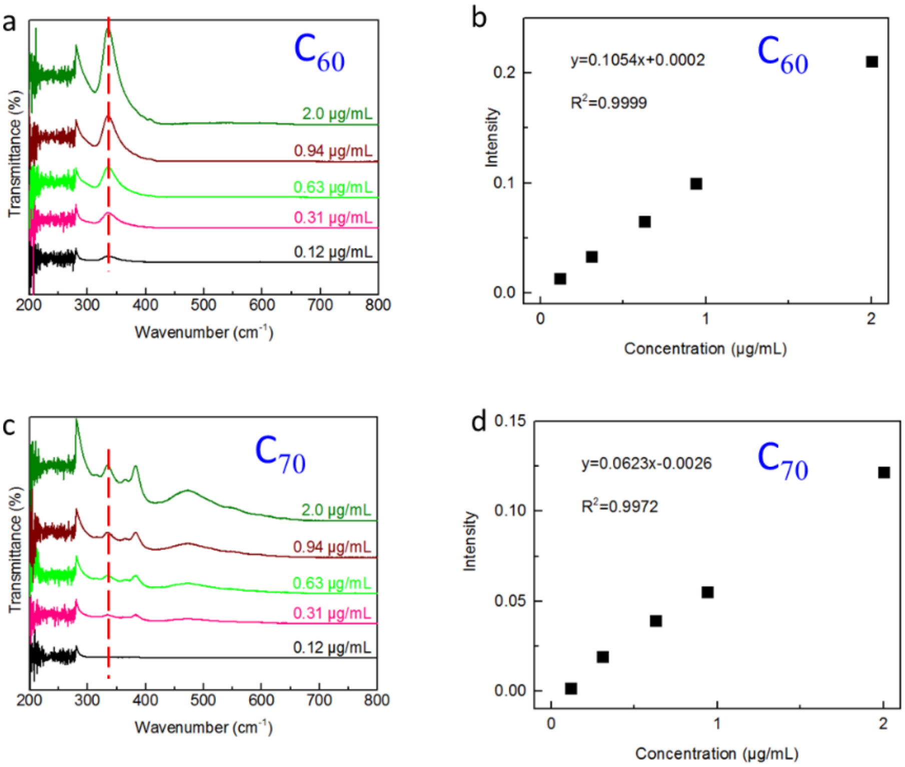

Figure S4. UV-vis standard curves of fullerenes in toluene.

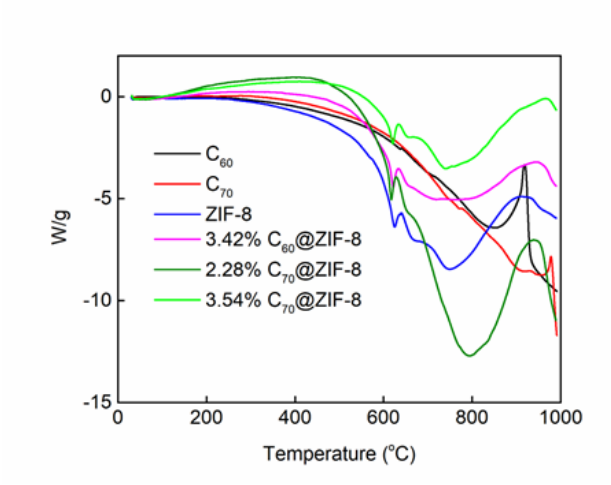

Figure S5. DSC curves of the fullerene@ZIF-8 nanoparticles.

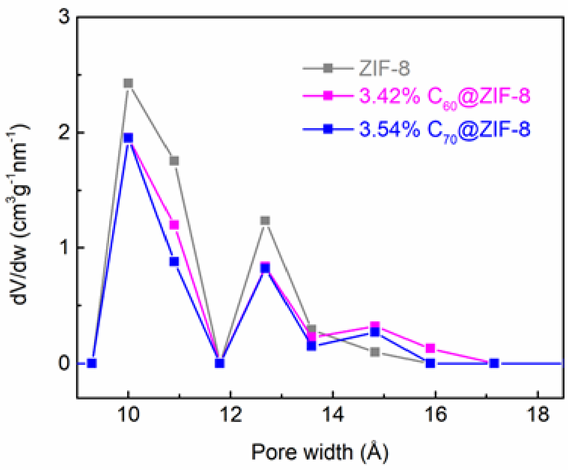

Figure S6. Pore size distributions of the fullerene@ZIF-8 nanoparticles from nitrogen adsorption.

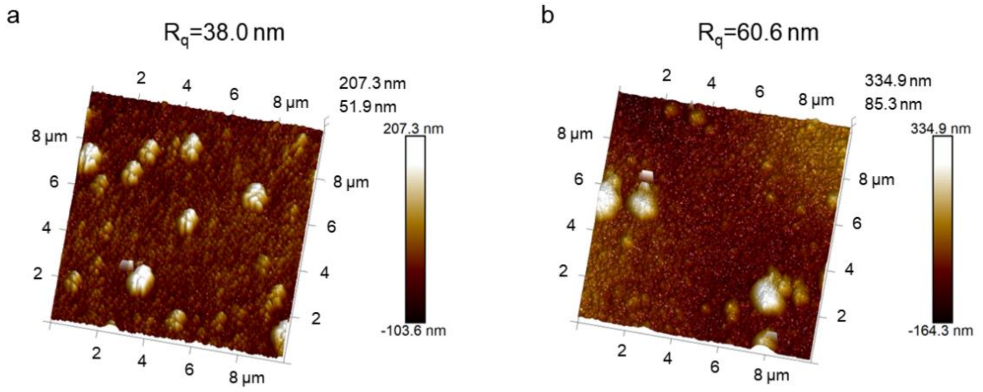

Figure S7. AFM images of the ZIF-8 (a) and 3.50% C_{70}@ZIF-8 (b) membranes.

Note: R_q: root mean square average of height deviations taken from the mean image data plane.

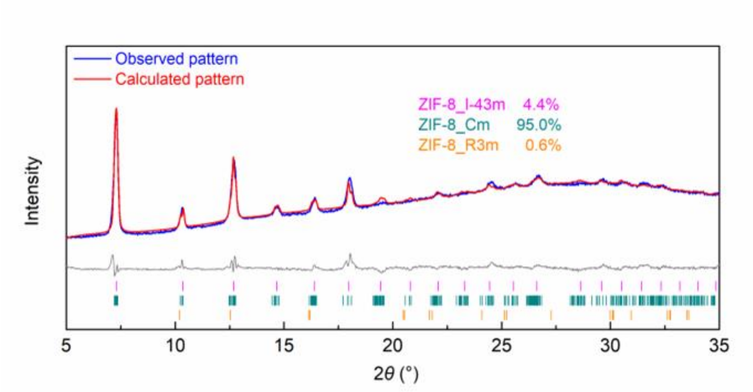

Figure S8. Rietveld refinement of the XRD result for the ZIF-8 membrane.

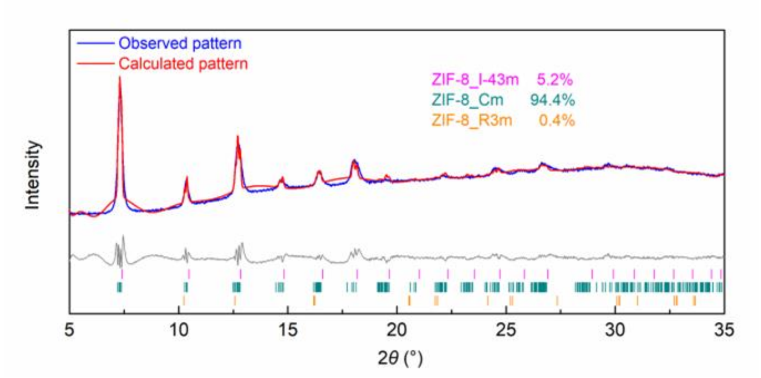

Figure S9. Rietveld refinement of the XRD result for the 3.63% C_{60}@ZIF-8 membrane.

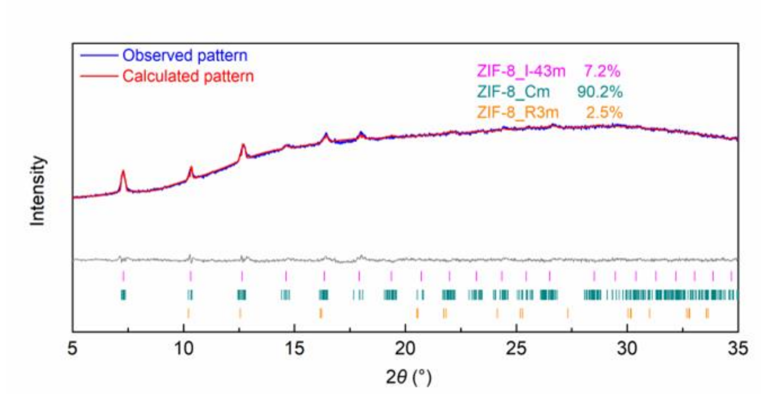

Figure S10. Rietveld refinement of the XRD result for the 2.42% C_{70}@ZIF-8 membrane.

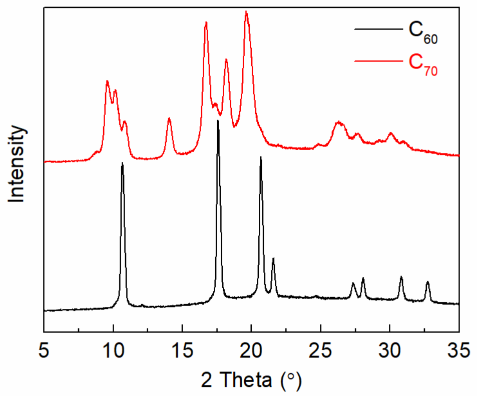

Figure S11. XRD spectra of the C_{60} and C_{70} powder.

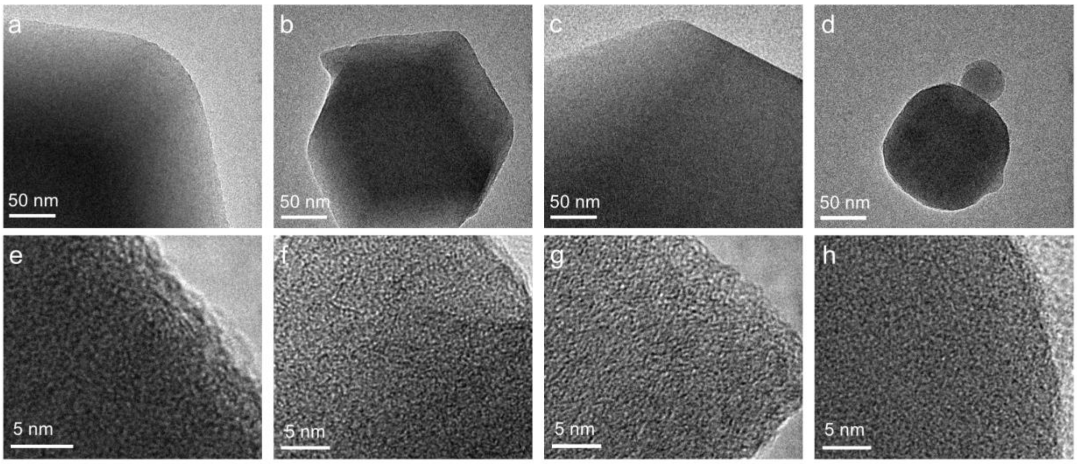

Figure S12. HRTEM images of ZIF-8 crystal (a, e), 3.54% C_{70}@ZIF-8 crystal (b, f), ZIF-8 membrane (c, g), and 3.50% C_{70}@ZIF-8 membrane (d, h).

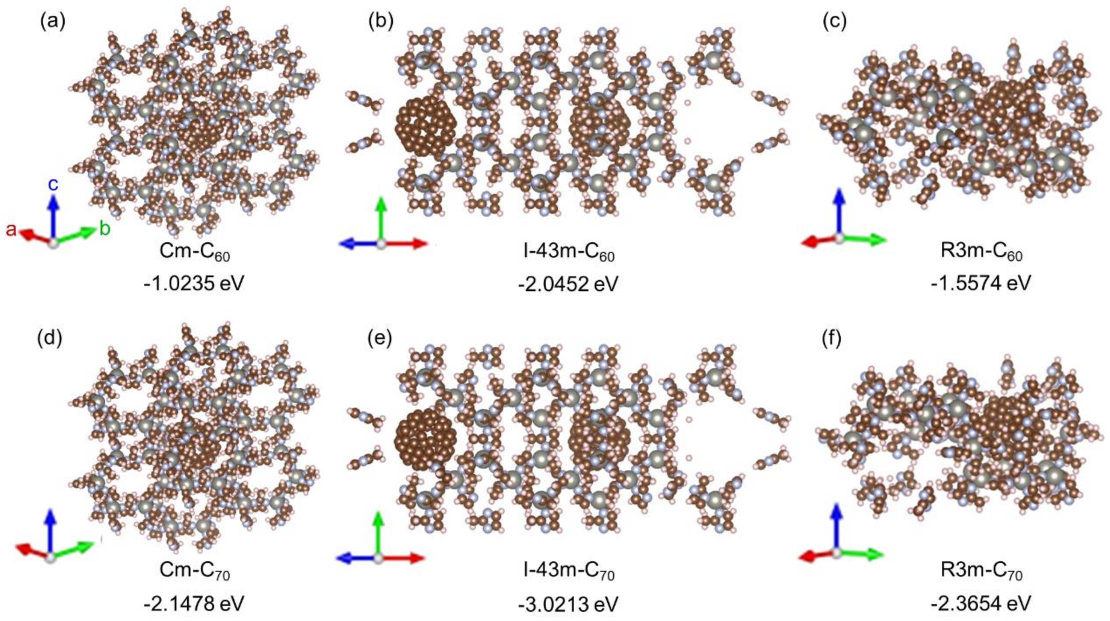

Figure S13. Interaction between fullerene and the different ZIF-8 phases.

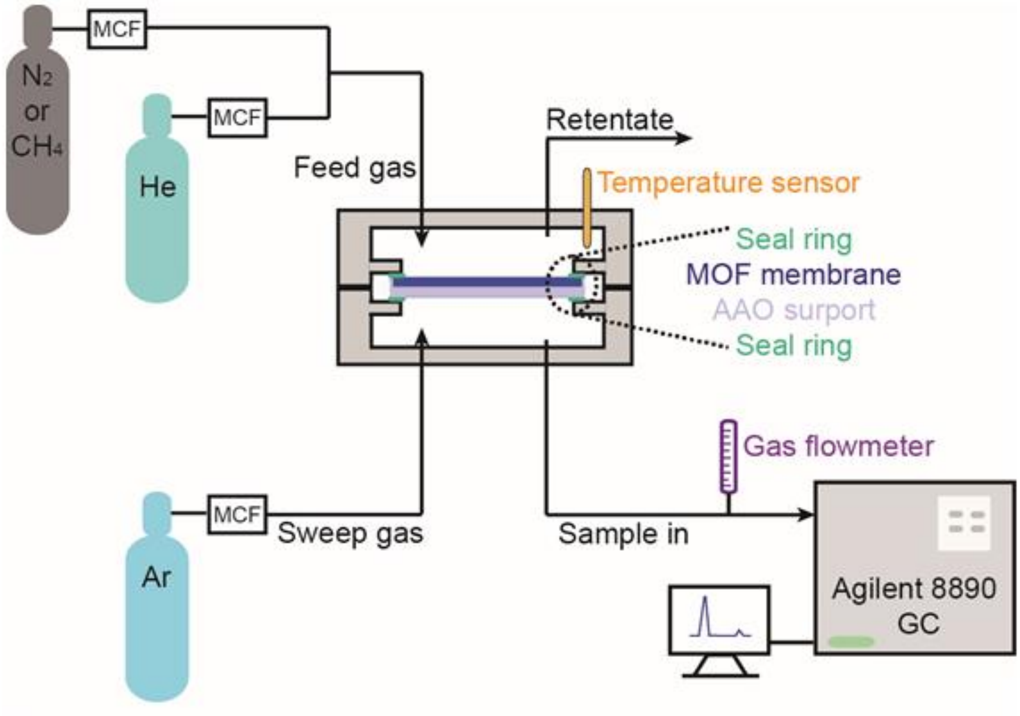

Figure S14. Schematic diagram of the test device of gas permeation based on Wicke-Kallenbach method.
Note: MCF: mass flow controller; GC: gas chromatograph.

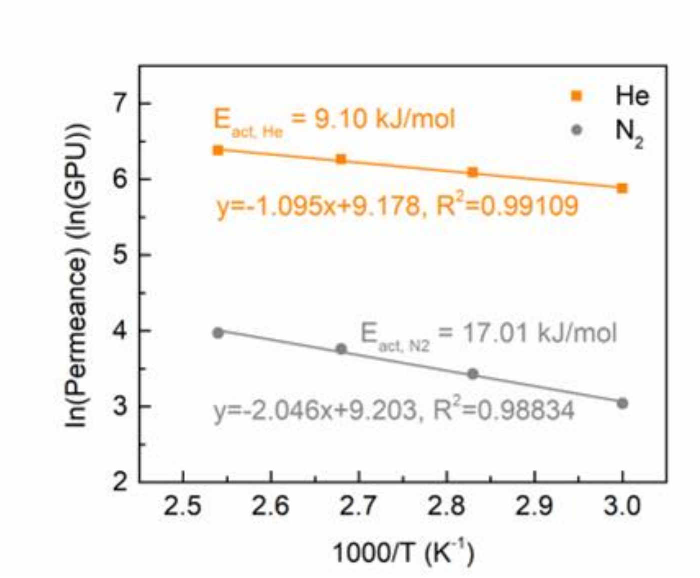

Figure S15. Arrhenius plots of He and N_2 permeances for the 3.50% C_{70}@ZIF-8 membrane.

Note:

The temperature dependence of gas permeation can be described by Arrhenius equation:

$$P_i = A_i \exp\left(\frac{-E_{\text{act, i}}}{RT}\right)$$

$$\ln(P_i) = \ln(A_i) - \frac{E_{\text{act, i}}}{R} \cdot \frac{1}{T}$$

where P_i is the gas permeance of component i, A_i represents the pre-exponential factor of component i, $E_{\text{act, i}}$ means the gas apparent activation energy of component i, R and T are the ideal gas constant (8.314 J mol^{-1} K^{-1}) and absolute temperature (K), respectively. A plot of ln(P) versus 1/T gives a straight line, whose slope is used to calculate $E_{act, i}$. The $E_{\text{act, He}}$ and $E_{\text{act, N2}}$ are 9.10 kJ/mol and 17.01 kJ/mol, respectively. This indicates a higher activated diffusion of N_2 than He.

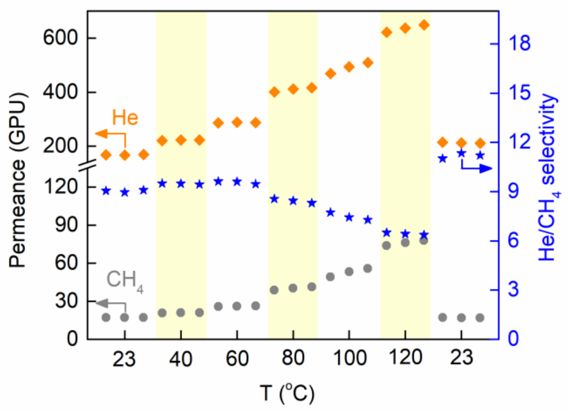

Figure S16. Effect of temperature on the He/CH_4 separation performance of the 3.50% C_{70}@ZIF-8 membrane.

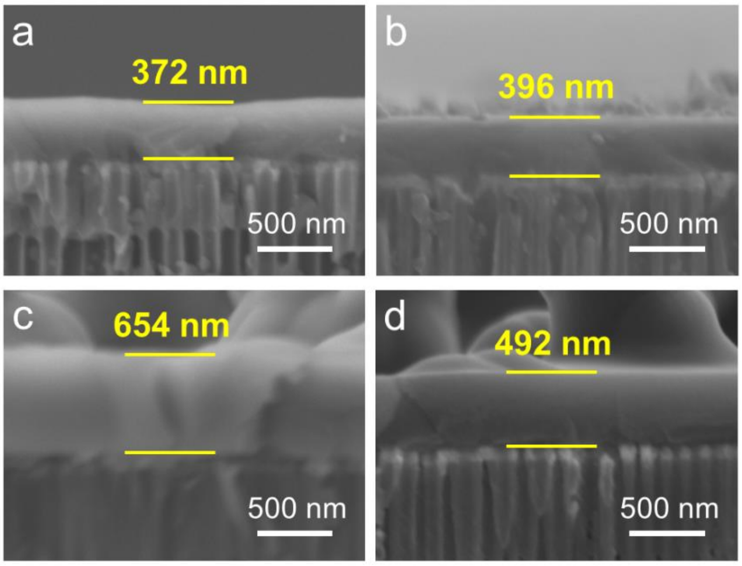

Figure S17. Cross-sectional images of the ZIF-76 (a), 3.79% C_{70}@ZIF-76 (b), ZIF-76-mbIm (c), and 3.54% C_{70}@ZIF-76-mbIm (d) membranes.

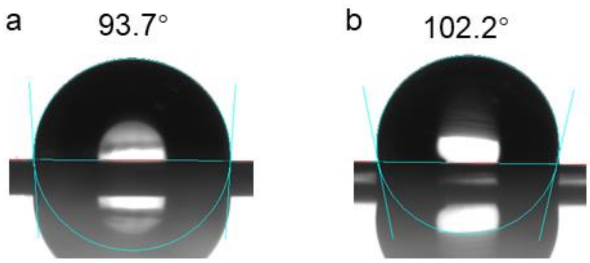

Figure S18. Water contact angles of the ZIF-76 (a) and 3.79% C_{70}@ZIF-76 (b) membranes.

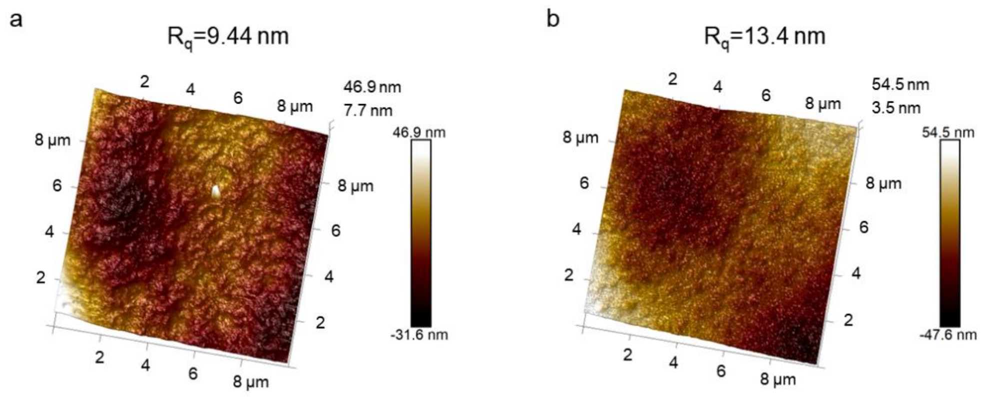

Figure S19. AFM images of the ZIF-76 (a) and 3.79% C_{70}@ZIF-76 (b) membranes.

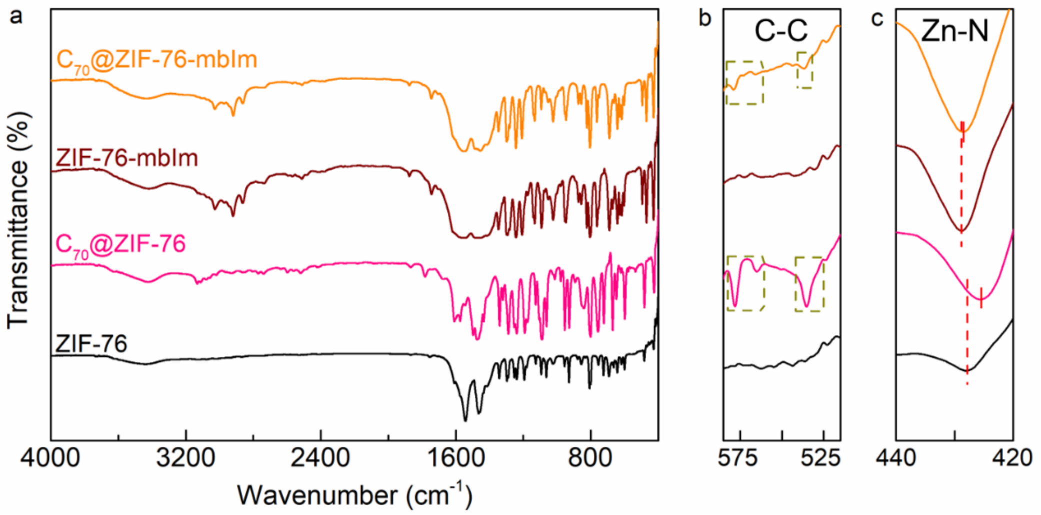

Figure S20. FTIR spectra of the ZIF-76, C_{70}@ZIF-76, ZIF-76-mbIm, and C_{70}@ZIF-76-mbIm nanoparticles.

Table S1. Dropping steps for preparation of the mother solutions.

Mother solutions	Dropping steps
600 μL C_{60}@ZIF-8	25 mL S-L + 300 μL S-C_{60} + 12.5 mL S-Zn + 300 μL S-C_{60} + 12.5 mL S-Zn
200 μL C_{70}@ZIF-8	25 mL S-L + 200 μL S-C_{70} + 25 mL S-Zn
400 μL C_{70}@ZIF-8	25 mL S-L + 400 μL S-C_{70} + 25 mL S-Zn
600 μL C_{70}@ZIF-8	25 mL S-L + 300 μL S-C_{70} + 12.5 mL S-Zn + 300 μL S-C_{70} + 12.5 mL S-Zn
800 μL C_{70}@ZIF-8	25 mL S-L + 400 μL S-C_{70} + 12.5 mL S-Zn + 400 μL S-C_{70} + 12.5 mL S-Zn
1000 μL C_{70}@ZIF-8	25 mL S-L + 400 μL S-C_{70} + 10 mL S-Zn + 200 μL S-C_{70} + 5 mL S-Zn + 200 μL S-C_{70} + 5 mL S-Zn+ 200 μL S-C_{70} + 5 mL S-Zn
1200 μL C_{70}@ZIF-8	25 mL S-L + 400 μL S-C_{70} + 5 mL S-Zn + 200 μL S-C_{70} + 5 mL S-Zn + 200 μL S-C_{70} + 5 mL S-Zn + 200 μL S-C_{70} + 5 mL S-Zn + 200 μL S-C_{70} + 5 mL S-Zn
1400 μL C_{70}@ZIF-8	25 mL S-L + 400 μL S-C_{70} + 5 mL S-Zn + 400 μL S-C_{70} + 5 mL S-Zn+ 200 μL S-C_{70} + 5 mL S-Zn+ 200 μL S-C_{70} + 5 mL S-Zn + 200 μL S-C_{70} + 5 mL S-Zn
1200 μL C_{70}@ZIF-76*	25 mL S-L + 400 μL S-C_{70} + 5 mL S-Zn + 200 μL S-C_{70} + 5 mL S-Zn + 200 μL S-C_{70} + 5 mL S-Zn + 200 μL S-C_{70} + 5 mL S-Zn + 200 μL S-C_{70} + 5 mL S-Zn
1200 μL C_{70}@ZIF-76-mbIm#	25 mL S-L + 400 μL S-C_{70} + 5 mL S-Zn + 200 μL S-C_{70} + 5 mL S-Zn + 200 μL S-C_{70} + 5 mL S-Zn + 200 μL S-C_{70} + 5 mL S-Zn + 200 μL S-C_{70} + 5 mL S-Zn

*: The ligand is composed of Im and cbIm with the molar rational of 9:1.

#: The ligand is composed of Im and mbIm with the molar rational of 2:1.

Table S2. Contents of fullerenes in the fullerenes@ZIF-8 nanoparticles and membranes.

Mother solutions	Mass content in ZIF-8 nanoparticles/wt%	Occupancy in ZIF-8 nanoparticles/%	Occupancy in membrane/%
600 μL C_{60}@ZIF-8	1.76	3.42	3.63
200 μL C_{70}@ZIF-8	0.52	0.84	1.40
400 μL C_{70}@ZIF-8	0.87	1.44	2.03
600 μL C_{70}@ZIF-8	1.38	2.28	2.42
800 μL C_{70}@ZIF-8	1.59	2.64	2.78
1000 μL C_{70}@ZIF-8	1.81	3.00	3.08
1200 μL C_{70}@ZIF-8	2.12	3.54	3.50
1400 μL C_{70}@ZIF-8	2.47	4.14	4.33

Table S3. Decomposition temperatures (T_d) of fullerenes, ZIF-8, and fullerene@ZIF-8.

Samples	T_d/°C *
C_{60}	734.1
C_{70}	775.0
ZIF-8	604.4
3.42% C_{60}@ZIF-8	530.8
2.28% C_{70}@ZIF-8	475.4
3.54% C_{70}@ZIF-8	512.4

*: The T_d values were obtained when the remaining weight was 95 wt% of the original weight.

Table S4. Comparison of the separation performance of this work with reported data.

Membranes	T/°C	L/μm	P_{He}/ GPU	$S_{He/N2}$	$S_{He/CH4}$	$S_{He/CO2}$	Ref.
Cu-BTC	-	-	4140	2.6	2.1	3.4	7
IRMOF-3	-	-	3046	2.5	1.61	1.7	8
IRMOF-3-AM6	-	-	2416	2.6	1.3	3.2	8
MMOF	25	-	41	3.0	-	2.8	9
MMOF	190	-	7.5	30	-	5.3	9
$[Cu_2(bza)_4(pyz)]_n$	20	-	7.9	3.9	7.3	0.29	10
MOF-5	25	14	896	2.4	-	2.9	11
ZIF-8	35	2.5	565	4.3	4.6	1.6	12
ZIF-8	25	80	126.6	4.7	4.0	1.6	13
3.50% C_{70}@ZIF-8	23	0.35	185.4	15.8	9.2	1.3	This work
3.50% C_{70}@ZIF-8	23	0.35	209.6	12.5			This work#
3.50% C_{70}@ZIF-8	23	0.35	65.5	30.4			This work*

#: Mixed gas separation performance.

*: Mixed gas separation performance under wet condition.

Table S5. He/N_2 and He/CH_4 mixed gas separation performance of the ZIF-76, C_{70}@ZIF-76, ZIF-76-mbIm, C_{70}@ZIF-76-mbIm membranes.

membrane	He/N_2		He/CH_4	
	$P_{He/\ GPU}$	$S_{He/N2}$	$P_{He/\ GPU}$	$S_{He/CH4}$
ZIF-76	77.6	5.7	79.8	4.6
3.79% C_{70}@ZIF-76	25.4	12.6	22.8	15.7
ZIF-76-mbIm	963	2.2	980	2.0
3.54% C_{70}@ZIF-76-mbIm	552	2.6	587	2.1

Table S6. Distances between different carbon atoms of ZIF-8_I-43m phase before and after C_{70} modification.

phase	d_8	d_1, d_6, d_7	d_9	d_5	d_2, d_4	d_3
ZIF-8_I-43m	4.48	5.23	5.96	6.66	7.21	9.93
C_{70}-ZIF-8_I-43m	4.43	5.20	5.94	6.56	7.47	9.93

References

(1) Zhou, S.; Wei, Y.; Li, L.; Duan, Y.; Hou, Q.; Zhang, L.; Ding, L.-X.; Xue, J.; Wang, H.; Caro, J., Paralyzed membrane: Current-driven synthesis of a metal-organic framework with sharpened propene/propane separation. *Sci. Adv.* **2018,** *4* (10), eaau1393.

(2) Zhou, S.; Shekhah, O.; Jia, J.; Czaban-Jóźwiak, J.; Bhatt, P. M.; Ramírez, A.; Gascon, J.; Eddaoudi, M., Electrochemical synthesis of continuous metal–organic framework membranes for separation of hydrocarbons. *Nat. Energy* **2021,** *6* (9), 882-891.

(3) Rappe, A. K.; Casewit, C. J.; Colwell, K. S.; Goddard, W. A., III; Skiff, W. M., UFF, a full periodic table force field for molecular mechanics and molecular dynamics simulations. *J. Am. Chem. Soc.* **1992,** *114* (25), 10024-35.

(4) Smit, B.; Maesen, T. L. M., Molecular Simulations of Zeolites: Adsorption, Diffusion, and Shape Selectivity. *Chem. Rev.* **2008,** *108* (10), 4125-4184.

(5) Zhang, L.; Hu, Z.; Jiang, J., Sorption-Induced Structural Transition of Zeolitic Imidazolate Framework-8: A Hybrid Molecular Simulation Study. *J. Am. Chem. Soc.* **2013,** *135* (9), 3722-3728.

(6) Thompson, A. P.; Aktulga, H. M.; Berger, R.; Bolintineanu, D. S.; Brown, W. M.; Crozier, P. S.; in 't Veld, P. J.; Kohlmeyer, A.; Moore, S. G.; Nguyen, T. D.; Shan, R.; Stevens, M. J.; Tranchida, J.; Trott, C.; Plimpton, S. J., LAMMPS - a flexible simulation tool for particle-based materials modeling at the atomic, meso, and continuum scales. *Comput. Phys. Commun.* **2022,** *271*, 108171.

(7) Cao, F.; Zhang, C.; Xiao, Y.; Huang, H.; Zhang, W.; Liu, D.; Zhong, C.; Yang, Q.; Yang, Z.; Lu, X., Helium Recovery by a Cu-BTC Metal-Organic-Framework Membrane. *Ind. Eng. Chem. Res.* **2012,** *51* (34), 11274-11278.

(8) Yoo, Y.; Varela-Guerrero, V.; Jeong, H.-K., Isoreticular Metal–Organic Frameworks and Their Membranes with Enhanced Crack Resistance and Moisture Stability by Surfactant-Assisted Drying. *Langmuir* **2011,** *27* (6), 2652-2657.

(9) Ranjan, R.; Tsapatsis, M., Microporous Metal Organic Framework Membrane on Porous Support Using the Seeded Growth Method. *Chem. Mater.* **2009,** *21* (20), 4920-4924.

(10) Takamizawa, S.; Takasaki, Y.; Miyake, R., Single-Crystal Membrane for Anisotropic and Efficient Gas Permeation. *J. Am. Chem. Soc.* **2010,** *132* (9), 2862-2863.

(11) Zhao, Z.; Ma, X.; Li, Z.; Lin, Y. S., Synthesis, characterization and gas transport properties of MOF-5 membranes. *J. Membr. Sci.* **2011,** *382* (1), 82-90.

(12) Liu, D.; Ma, X.; Xi, H.; Lin, Y. S., Gas transport properties and propylene/propane separation characteristics of ZIF-8 membranes. *J. Membr. Sci.* **2014,** *451*, 85-93.

(13) Hara, N.; Yoshimune, M.; Negishi, H.; Haraya, K.; Hara, S.; Yamaguchi, T., Diffusive separation of propylene/propane with ZIF-8 membranes. *J. Membr. Sci.* **2014,** *450*, 215-223.
